# Inflammation, infection and coagulation disorders

**DOI:** 10.1097/HS9.0000000000000267

**Published:** 2019-06-30

**Authors:** Per Morten Sandset

**Affiliations:** 1 University of Oslo, Oslo, Norway; 2 Oslo University Hospital, Oslo, Norway

**Learning goals**

- Understand the most important interactions between inflammation and coagulation.
- Understand the mechanism(s) and role of NETs formation.
- Understand the pathophysiology of DIC.
- Understanding and knowledge of correctional treatment of different coagulopathies.

## Introduction

There is now growing evidence for a crosstalk between inflammation and coagulation. In this session, Konstantin Stark report on the current evidence for a crosstalk between platelets and neutrophils. These cells are among the first responders to pathogens and perturbation of vascular integrity and the interplay triggers neutrophil extracellular trap (NET) formation. NETs have many functions including providing a scaffold for platelet adhesion and enhanced platelet activation. Cheng-Hock Toh provides an update on the pathophysiology of disseminated intravascular coagulation (DIC), a condition characterized by severe and uncontrolled activation of coagulation that may lead to multiorgan failure secondary to thrombosis in the microvasculature and in medium sized vessels and also due to consumptive coagulopathy leading to bleeding. Novel aspects of pathophysiology include mechanisms of thrombin generation, cellular dysfunction in the microcirculation and the contribution of innate immunity and inflammation with NETs formation. Finally, Riitta Lassila discusses management of coagulopathies in a selection of severe inflammatory disorders.

